# Biomechanics of Atlas Cedar Roots in response to the Medium Hydromechanical Characteristics

**DOI:** 10.1155/2020/7538698

**Published:** 2020-08-18

**Authors:** Belkacem EL Amrani, Mohammed Bendriss Amraoui

**Affiliations:** Laboratory of Biotechnology, Environment, Food and Health (LBEFH), Department of Biology, Faculty of Sciences Dhar el Mahraz, Sidi Mohammed Ben Abdellah University, P.O. Box 1796, Atlas, Fez, Morocco

## Abstract

The biomechanical root flexibility in response to hydromechanical soil heterogeneity is the most determining factor of the root architecture which plays a paramount role in mycorrhizal infection and allows the seedlings to adapt to the environmental constraint. We examined the impact of five different hydromechanical medium properties (hydroponics, vermiculite, vermiculite-gravel, sawdust, and sand) on the morphology, physiology, and anatomy of *Cedrus atlantica* seedlings at a controlled growth chamber. The growth of the seedling is strongly stimulated by the hydroponic medium through the stimulation of the aerial part dry weight and the main root length. However, the sand medium increases the main root dry weight by the radial expanse stimulation at the level of the epidermis, vascular cylinder, and cortex and compensates the less root architecture by the stimulation of the xylem and phloem areas. In contrast to sand and hydroponic media, the sawdust medium stimulates the phloem/xylem ratio, the root architecture, and the short roots. The Pearson bilateral correlation shows that the aerial part dry weight is positively correlated with the permeability, porosity, and water-holding capacity and negatively with the bulk density and density at saturation, whereas the short root production is negatively correlated with the permeability and water-holding capacity. Hence, the hydromechanical characteristics of the soils must be taken into account in the reforestation and mycorrhization attempts.

## 1. Introduction

The growth of the forest seedlings is conditioned by many abiotic factors, in particular the physics of the soil and its hydrological status [1]. They make the seedling dependent on the optimal root architecture [2, 3] which allows detection and response to environmental challenges through the growth change [4–6]. Moreover, it has been reported that the shoot and the total seedling growth are related to the performance/adaptation of the root system, which is in direct contact with the variation of the hydromechanical soil properties. Therefore, the root system growth is crucial for seedling survival and forest regeneration. This ascertainment agrees with the studies performed by Zha et al. [7] and Yan et al. [8] who have demonstrated that the developmental mechanisms, which regulate root response to the soil mechanical forces, play a central role in the development of the shoots and plant survival.

Although its reaction to several constraints has been studied little, *Cedrus atlantica* (Endl.) Manetti ex Carrière is a model plant of the Mediterranean forests. It plays an important economic and ecological role and is a species candidate for afforestation in the Mediterranean region [9, 10]. Additionally, its natural regeneration and transplantation are actually exposed to many obstacles that are related to biological, chemical, and, especially, physical soil qualities. In addition to these natural obstacles, the cedar seedlings are challenged by the early slow growth [11] and the weak mycorrhization [9, 12, 13]. All previous trials to *Cedrus atlantica* mycorrhizal inoculation prove that the failure of mycorrhizal development is related to the low root receptiveness and the slow development of the lateral root and the fewer availability of fine roots [9, 13, 14]. Some of the authors concluded that the difference in the infection rate and the low number of roots susceptible to infection result from the medium effect [13]. Other authors confirm that the fine root formation can be increased by the culture medium [9]. Recently, a progress has been made on the response of Atlas cedar to the natural soil texture [15], but our knowledge remains still imperfect on the effect of the physics of the soil and its hydrological status on growth strategies of the root architecture. In the same way, it was reported, in a wide range of species, that the root architecture development depends on soil mechanical properties [16]. Similarly, it has been showed that the mechanical impedance is the major reason that causes the poor root system growth and development [17]. For example, the root elongation is more controlled by the soil mechanical properties rather than chemical ones [18]. The soil hydraulic properties also play an important role in determining the root system architecture [19, 20]. However, in natural conditions, the soil hydraulic properties depend mainly on the mechanical and textural characteristics, and several studies have shown a strong relationship between them. For example, it has been found that the water-holding capacity and permeability enormously take into account the soil density [21] and soil porosity [22] and also depend on the presence of organic matter and gravel [23]. In our opinion, the extent of compaction which is usually evaluated in terms of soil density, porosity, and root penetration rate [24] may also affect the root architecture.

For a maximal success of the afforestation and the mycorrhization of cedar seedlings, we still need to learn more about its root system architecture and the ways to control its growth and development. Therefore, we need to understand the hydrological and mechanical medium factors that increase the roots' receptiveness toward mycorrhizal fungi. On this basis, we have studied the growth response of *Cedrus atlantica* seedlings to the variation of the medium hydromechanical characteristics through their morphological and anatomical root traits.

## 2. Materials and Methods

### 2.1. Seedling Growth Conditions

About thirty mature (semiopen) Atlas cedar cones, containing an average of 115 seeds, were harvested in September 2017 from a sunny tree at the edge of the Moroccan cedar forest of Saheb (33°22′4″N; 5°13′23″W; 1,824 m). The cones' scales were removed, and seeds were collected and sorted afterwards according to their size and weight. Thereafter, the homogeneous seeds were selected and immediately stratified at 4°C for 15 days. The seeds stratified were soaked for 48 h and then transferred on a wet paper until the appearance of a radicle of about 1.5 cm. The germinated seeds were maintained separately (five seedlings per culture medium with one seedling per pot) into pots of 07 × 07 × 25 cm containing a medium with different mechanical properties (hydroponic: Hyd, vermiculite: Ver, vermiculite-gravel: VGr (2/1, V/V), red sawdust: Saw, and sand: San) (Table 1). All the seedlings were fertilized weekly with a nutrient solution composed of 5.0 mM Ca (NO_3_)_2_; 1 mM KCl; 0.2 mM CaCl_2_; 0.2 mM KH_2_PO_4_; 0.1 mM MgSO_4_; 0.5% FeCl_3_; 0.2 mL L^−1^ of a trace element solution [25], pH 5.5. The cultures were maintained for 90 days in a controlled growth chamber under a temperature ranging from 24°C to 32°C, a photoperiod of 16/8 h, a light intensity of 70 *µ*mol m^−2^ s^−1^, and a relative humidity of the order of 50 to 70%. All seedlings were daily moistened with water during dry periods.

### 2.2. Characteristics of Culture Media

The mechanical and hydraulic properties of culture medium were characterized by (1) permeability (K, cm s^−1^), which was determined according to Darcy's law by the equation (*Q* × *L*) ÷ (*t* × *A* × *h*)  where *Q* is the volume of water collected (mL), *L* is the sample height (cm), *t* is the duration of water collection (sec), *A* is the cross-sectional area of the medium (cm^2^), and *h* is the total constant head (cm); (2) porosity (*n*), which is determined as the ratio of the volume of voids to the total volume of the medium; (3) bulk density (*ρ*_*d*_, g cm^−3^), which is determined as the dry weight of solid particles per unit volume [26]; (4) density at saturation (*ρ*_sat_, g cm^−3^), which is determined as the weight of solid particles plus water per unit volume; (5) water-holding capacity (WHC, %), which is calculated according to the European method as the percentage of water held in the sample (V/V) [27].

The hydroponic medium significantly possesses the maximum values of the permeability, porosity, and water-holding capacity and the minimum values of the bulk density and density at saturation compared to the sand and vermiculite-gravel media. However, the vermiculite and sawdust media significantly display intermediate values of all hydromechanical properties compared to the others, except between hydroponic and sawdust media in density at saturation and between sawdust and vermiculite-gravel media in water-holding capacity (Table 1).

### 2.3. Data Analyzing

The growth was estimated on four repetitions by weighing the dry mass of needles, stem, main root, lateral roots, and short roots obtained after drying at 70°C for 48 h. Water content was estimated by the difference between the matter fresh weight (FW) and dry weight (DW) of each seedling organ and calculated according to the equation (FW − DW) × 100 ÷ (DW) . Root branching was estimated by measuring the length and the number of different root categories using the ImageJ software (Version 1.50i, 2016). The root density is determined as the ratio of the lateral root number to the main root length. The mean value of the root penetration rate of cedar seedlings was determined as the ratio dl/dt where dl is the variation of the length of the main root (mm) and dt is the variation of time (day) from sowing the sprouted seeds in a pot until the seedling is harvested.

The most important anatomical characteristics of roots such as the width of the epidermis, vascular cylinder, and cortex tissues, the diameter of the main root, and the diameter and the number of the cortex cells were performed by freehand transverse cross sections at 25 to 35 mm behind the root apex [5, 28]. Afterwards, the preparations were immediately photographed by using the integrated camera of a light microscope (Optika DM-15). The best five cross sections were selected and used to determine the anatomical characteristics for each seedling. Because the root is not perfectly circular under the effect of the solid particles in the medium, the number of cortex cells and the width and the diameter of the tissues were evaluated on ten different places of each cross section, while the area was measured once using the ImageJ software (Version 1.50i, 2016). The mean value of the cortex cell diameter was calculated as the ratio of the width of the cortex to the cell number of the cortex.

### 2.4. Statistical Analyses

The obtained data have been subjected to the analysis of variance (ANOVA), and the means were compared by Fisher's least significant difference (LSD) post hoc test at *P* < 0.05. The Pearson bilateral correlation coefficients were calculated at 5% risk of the error. All these statistics have been done by using IBM SPSS Statistics (Version 20.0, 2011).

## 3. Results

### 3.1. Seedling Growth

**Figure 1:**
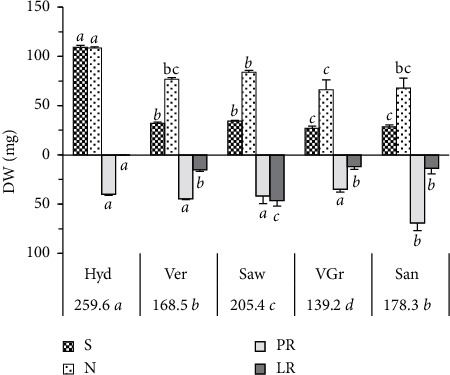
Dry weight (DW) average of cedar seedlings in response to the medium variation. *N*: Needles, *S*: stem, PR: main root, and LR: lateral roots. The values at the base of the histograms indicate the average DW of the seedlings. Letters indicate significant differences (*P* < 0.05 LSD test). The acronyms of media are as designated as in Table 1.

The 90-day-old seedlings showed the greatest dry weight on the hydroponic medium and the lowest on the vermiculite-gravel mixture. The hydroponic medium significantly stimulates the growth of needles and the stem (*P* < 0.009), whereas the lowest values are observed at the vermiculite-gravel and sand media. The root system growth shows a different behavior from that of the shoots, so that the sand, compared to the other media, significantly stimulates (*P* < 0.002) the main root with an average of 69.43 mg. The dry weight of the lateral roots is significantly maximal (*P* < 0.0001) and minimal (*P* < 0.023) on the sawdust and hydroponic media, respectively (Figure 1).

### 3.2. Hydrological Status of Seedlings

The water content of cedar seedlings largely depends on the mechanical strength caused by the nature of the medium used. The hydroponic medium decreases the water content of the seedling especially at the level of the stem (*P* < 0.0001) with an average of 2.18% against 125.49% as the lowest value on the other media. Similarly, it significantly decreases (*P* < 0.0001) the water content of the lateral roots to 13.64%, whereas the maximum is observed on the sawdust and vermiculite with 457.03 and 436.58%, respectively. On the other hand, at *P* < 0.028, sawdust and vermiculite significantly decrease the water content of the needles, while the hydroponic increases the water content of the main root (Table 2).

### 3.3. Seedling Root Architecture

**Figure 2:**
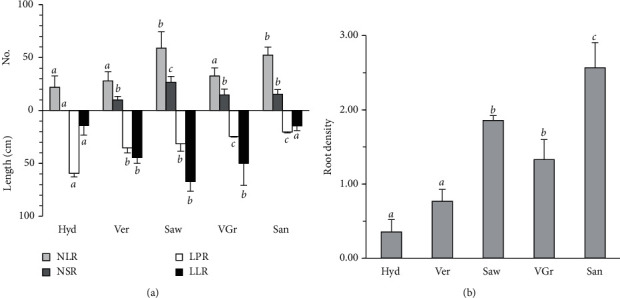
Root architecture of *Cedrus atlantica* Manetti in response to the variation of the substratum hydromechanical constraint. (a) Number of lateral roots (NLR) and short roots (NSR); length of the main root (LPR) and lateral roots (LLR). (b) Lateral root density. Letters indicate significant differences (*P* < 0.05 LSD test). The acronyms of media are as designated as in Table 1.

**Figure 3:**
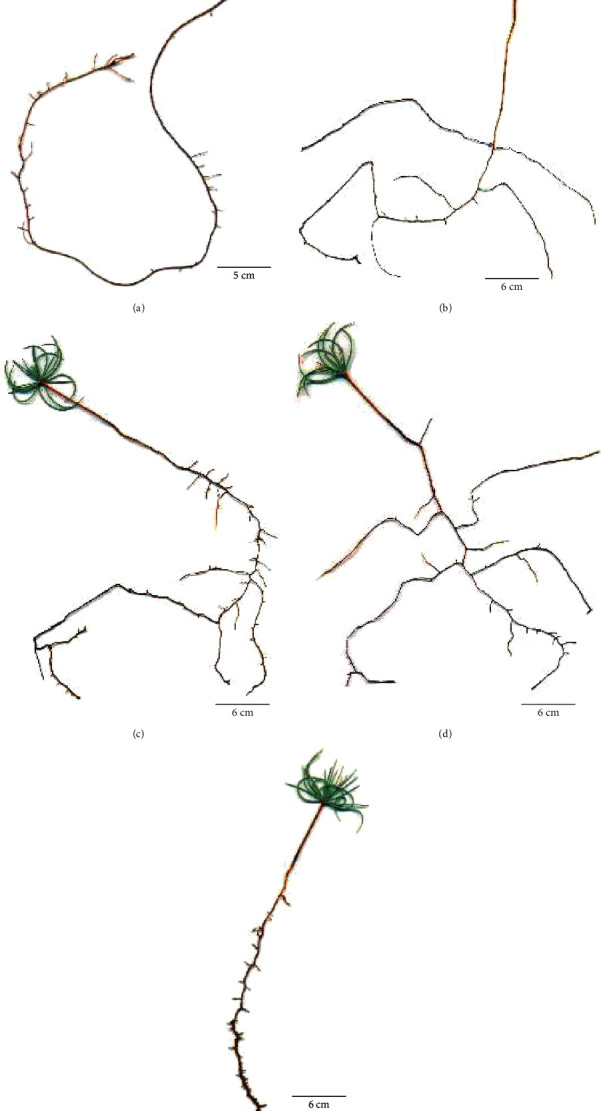
Root system architecture of cedar seedlings growing on five media with different hydromechanical constraints: (a) Hyd, (b) Ver, (c) Saw, (d) VGr, and (e) San. The scale bar corresponds to 5 cm.

The hydroponic significantly (*P* < 0.0001) stimulates the main root length (59.47 cm) compared to all media and strongly reduces the lateral root number compared to sawdust and sand and the lateral root length compared to vermiculite, sawdust, and vermiculite-gravel media (Figures 2(a) and 3). The average abundance of short roots (<0.5 mm) is the maximum on sawdust and minimum on the hydroponic medium (Figures 2(a) and 3). A high root density is observed on the sand with 2.57, while a low one is noted on the hydroponic with 0.36 (Figure 2(b)).

### 3.4. Anatomical Characteristics of Root

The root anatomy of cedar seedlings shows that sand and vermiculite, respectively, increase and decrease the main root and the cortex cell diameters and the width of the epidermis, vascular cylinder, and cortex tissues (*P* < 0.0001) compared to the other media except between hydroponic and vermiculite for the cortex cell diameter and the cortex width. The cortex tissue is also stimulated in the number of cells by the sand medium (*P* < 0.012). However, the stimulation of the sawdust, hydroponic, and vermiculite-gravel media is statistically lower than sand's (Table 3).

### 3.5. Vascular Bundle of Root

**Figure 4:**
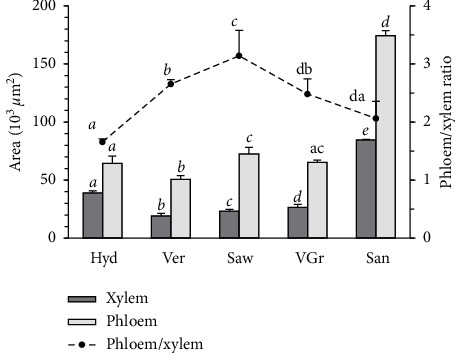
Phloem and xylem characteristics of the cedar main root. Letters indicate significant differences (*P* < 0.05 LSD test). The acronyms of media are as designated as in Table 1.

The measurement of the vascular bundle area at the cedar main root shows that the sand significantly (*P* < 0.0001) increases the xylem and phloem areas with 84.55 × 10^3^ and 174.27 × 10^3^ *µ*m^2^, respectively, compared to the other media. Moreover, the xylem and phloem areas are, respectively, broad and narrow on hydroponic compared to the sawdust. Although the hydroponic and sand have extreme mechanical strength (low and high, respectively), they reveal a statistically low value of the phloem/xylem ratio. In contrast, the sawdust which has an intermediate mechanical strength reveals a maximum value of the phloem/xylem ratio with 3.14 (Figure 4).

A gradual decrease in the penetration ability was observed from hydroponic to the sand medium in response to the increase and the decrease in the medium strength and hydraulic properties, respectively. The hydroponic medium shows a high penetration rate of 0.661 mm/day against 0.226 mm/day for the sand medium with a significant difference at *P* < 0.0001 (Table 4).

## 4. Discussion

This study shows that the nonexistence of mechanical impedance combined with the high availability of water in the hydroponic environment improves the growth of the aerial part of cedar seedlings (Figure 1 and Table 1). However, the continuous and partial mechanical barriers of the sand and vermiculite-gravel media (sand particle and gravel, Table 1) reduce its aerial part growth and the length of its main root (Figures 1 and 2(a)). This suggests that the cedar shoot growth is highly dependent on the hydromechanical medium properties. The significant correlation found here between the aerial part growth and all the hydromechanical proprieties (positively with K, *n*, and WHC and negatively with *ρ*_d_ and *ρ*_sat_) supports this conclusion (Table 6). In accordance with this, previous study findings have shown that dry matter of shoots [29] and leaf expansion [17] decrease in the hard soil treatments. Moreover, Cambi et al. [24] have reported that a higher soil compaction reduces the seedling performance in terms of height and root growth. Furthermore, the decrease in the shoot growth in response to the increases in medium hardness could be a result of the inhibition of CO_2_ diffusion to the mesophyll and the reduction of the transpiration and photosynthesis rate as revealed by several studies [24, 29–31]. According to these studies, the reduction in transpiration and its consequences are induced by the direct abscisic acid signaling, between the root and the shoot [32], that is largely involved in the responses of the plant growth to the compaction and water drought [33, 34].

Although the hydroponic medium stimulates the main root length, its dry weight remains statistically unchanged compared to the other media (Figures 1 and 2(a)). Though the main root length decreases, its dry weight and diameter increase on the sand medium (Figure 2(a)). This agrees with previous studies which show that the reduction of the root elongation is associated with radial expansion [3, 35]. The dissimilarity of the main root length and diameter responses to the different media was evident and suggests that these two growth parameters depend on different hydromechanical properties. A correlation of these properties with the length and the diameter of the main root was found in the opposite direction between these growth parameters and confirms this suggestion. For example, the length is positively correlated with the permeability, porosity, and water-holding capacity, while the diameter is negatively correlated with the two latter parameters and vice versa for the bulk density and density at saturation (Table 6). This finding supports previous research studies on different hardwood species of *Quercus* genus [1, 24, 36], conifer species [37], and other species [18] which show that the length and the diameter of the main root decreases and increases, respectively, in response to the high soil density and compaction.

It was shown that the deeper root system and the root penetration ability guarantee the acquisition of water and nutrients for the plant [8, 38, 39], and thus the very shallow and narrow root system is effectively decreasing the soil exploration rate [18]. The effect of the increase in the medium strength and the decrease in the hydraulic properties on the root penetration rate of cedar seedlings was found to be strong (Tables 1 and 4). The reduction in the root penetration in relation to that in hydroponic vary from 1.7 to 2.9 times according to the medium. Such an experimental result had suggested that the decreases in the main root length can be resulted from a reduction in the elongation zone size [40]. Another study has reported that the penetration rate depends on the pressure originated primarily from the extension of cells located in the elongation zone of the main root tip [41] or even from the interruption of meristematic zones activity [3]. Moreover, it was announced that the mechanical impedance alters auxin and ethylene responses in the root but increases endogenous abscisic acid, leading to the enlarging of the root diameter [28, 34]. According to these data, we can argue that (i) mechanical barriers and hydraulic property of the medium alter auxin and ethylene response in cedar roots or (ii) the cell extension in the cedar root elongation zone did not develop sufficient pressure to counteract high compaction due to the low auxin gradient in the root apical meristem [42]. This assumption is confirmed by the high decreases in the main root extension (59.47 to 35.44 cm), developed by the seedlings, when the bulk density (*ρ*_*d*_) has been increased slightly from 0.000 to 0.349 g cm^−3^. Moreover, when bulk density gradually increases, the root penetration rate decreases from 0.661 to 0.226 mm/day (Figure 2(a) and Tables 1 and 4). The weak auxin gradient in the cedar apical root is also proven by the strong extension of the lateral root when the main root encounters an obstacle whose power is equal to or greater than 0.349 g cm^−3^.

At the anatomical scale, the high compaction of the sand induces a high mechanical impedance (high *ρ*_d_ and *ρ*_sat_) and increases all tissue width and number, and thus the main root diameter is mainly through the width of the cortex and vascular cylinder (Tables 1 and 3). This makes the roots thicker and more resistant to the penetration of strong media as reported by Jin et al. [41] and Clark et al. [43]. These findings agree with those provided by Chimungu et al. [5] who show that the higher tissue density improves rigidity and strength to increase the root penetration ability in strong soils. Furthermore, the vermiculite medium which was found to significantly reduce the diameter of the main root at the level of the width of the epidermis and the vascular cylinder compared to the other media did not affect the width of the cortex at the level of the diameter and the number of cortex cells compared to hydroponic alone (Table 3). On the other hand, the vermiculite-gravel and sawdust which were found to increase the diameter of the main root at the level of the cortex cell diameter and the width of the cortex did not affect the number of cortex cells and the width of the epidermis and vascular cylinder compared to the hydroponic (Table 3). These variations of the main root diameter at the level of the epidermis, the vascular cylinder, and the cortex width, according to the degree of medium compaction, show that the Atlas cedar adjusts its root penetration mainly by the cortex and the vascular cylinder area in the hard medium and by the epidermis and the vascular cylinder area in the nonhardy medium. Moreover, the high correlation of the width of all studied root tissues (epidermis, vascular cylinder, and cortex) with all hydromechanical proprieties confirms this conclusion (Table 5). These data reflect the findings which show that the root's thickness in high-strength soils coincides with the increase in the cortical area at a wide range of species [6, 44]. This is consistent with the important role of the thick cortex region in stabilizing the root and reducing the metabolic cost in the cell turnover at the root cap to explore soil layers during the penetration as it has in cedar seedlings [5, 45].

**Figure 5:**
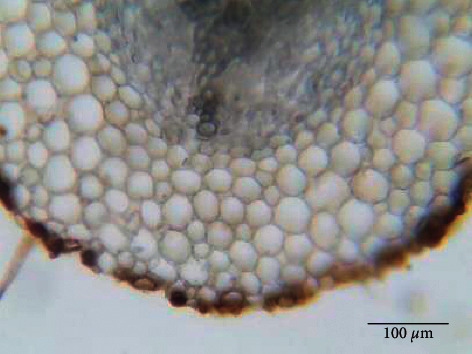
Main root cross section of the cedar seedling grown on the hydroponically medium. The scale bar corresponds to 100 *µ*m.

The water saturation of the hydroponic medium needs larger conductor vessels in Atlas cedar to transport and purge excess water at the root level through the transpiration. This explanation is consistent with the absence of aerenchymas in the presence of excess water as it is shown in Figure 5.

Sand medium significantly increases the xylem and phloem areas (Figure 4). According to Whalley et al. [46] and Jung et al. [47] studies, this increment aims to increase the water and the mineral salts up to the shoot to compensate the weak root branching and the low ability of cedar seedlings to explore a larger soil volume (Figures 2(a) and 3 and Table 4). Moreover, the sand medium decreases the phl/xyl ratio to promote the rise of the raw sap to the detriment of the elaborate sap as it was found in the case of the hydroponic medium (Figure 4). This anatomical strategy suggests that under these two media, the cedar seedlings tend to maintain the growth of the aerial part. For it, we find that under these two media, the cedar seedlings have a similar and significantly high water content at the needles (Table 2). This is in conformity with the findings by Hameed et al. [48] who show that the seedlings increased the mesophyll thickness as a strategy to drought tolerant because this would help stabilize photosynthesis [49] as has been found here for the root cortex width. On the contrary, the sawdust medium stimulates the phl/xyl ratio (Figure 4). This action aims to favor the transport of the elaborated sap toward the roots in order to meet carbon requirements and support the stimulation of lateral and short root growth (Figures 1 and 2(a)). This explanation is in agreement with the data of sawdust which preserves the water level of the lateral roots by reducing water use in photosynthesis, as it has sustained by the low water content of the needles and the high water content and growth of the lateral roots (Figures 1 and 2(a) and Table 2). In sum, these data suggested that the vascular bundle may better explain the variation in cedar seedling growth strategies in response to the soil hydromechanical conditions.

The sawdust which is organic at 100% and characterized by a low density (Table 1) stimulates the dry weight and the number and length of the lateral roots and short roots (Figures 1 and 2(a)). In contrast, sand significantly stimulates root density but reduces considerably the length of the lateral root (Figures 2(a) and 2(b)), which leads to a poor root architecture on this soil. Moreover, the lateral roots are almost absent on the hydroponic medium (Figures 1 and 2(a)). The absence of the mechanical stress and the presence of excess water give birth to a very long main root with few lateral roots which lead to a less root architecture, and consequently, a less formation of the short root, given that the lateral roots are the major determinants of the cedar root architecture as reported by Szymanowska-Pułka [3] for many species. Even if the strong mechanical stress with a low permeability and a low water-holding capacity (case of sand) stimulates the lateral root formation of seedlings, these roots do not elongate and do not develop into a root architecture because of the low abundance of short roots. These results agree with several studies which reported that the mechanical properties of the medium affect the root system architecture [8, 16, 44].

The present results show that the medium rich in organic matter (sawdust), followed by those composed of vermiculite, is optimal for the formation of a better root architecture (lateral and short roots), while purely hydrous or sandy media are the worst (Figures 1 and 2). In addition, a close relationship is found among the root branching (lateral and short roots) and the permeability and water-holding capacity (Table 6), which is in accordance with the data by Jung et al. [47] who have showed that the decline in the fine root is due to the lack of water capillary rise and the compaction. These data also agree with previous studies which reported that the increase in soil density impedes the lateral root extension [21] and reduces particularly the short root number like it has been found in *Quercus petraea* Liebl. seedlings [1]. Furthermore, the vermiculite medium, which has intermediate values of permeability, porosity, and water-holding capacity between the hydroponic and the sawdust media (Table 1), gives rise to a root architecture significantly lower in terms of the number of lateral and short roots than the sawdust (Figure 2(a)). This confirms why the sawdust is an optimal condition for lateral and short root production and may also suggest that not only the optimal hydromechanical properties, but also the abundance of organic matter are the two elements necessary for the formation of lateral and short roots that are the headquarters of mycorrhization which may facilitate the receptivity of cedar species to the ectomycorrhiza fungi, and thus to the seedlings' survival.

The difference between the change in relative seedling and adult abundance confirms that the Atlas cedar forests are waning more and rejuvenated less nowadays [50, 51]. In addition to the anthropic activities, the *C. atlantica* decline is largely related to the climatic and edaphic conditions. It has proved that drought affects dramatically the hydraulic conductivity and the xylem vulnerability of this species [52]. Moreover, *C. atlantica* distribution is strongly depending on the soil nature so that the carbonatic soils are tolerated only when the soil is deep and stations are sufficiently rainy [53] or rather when the seedlings are mycorrhized [54]. From our results, we can suggest that the fight against the regeneration difficulties and afforestation program failure requires the improvement of the growth/performance of the cedar seedling through the adjustment of the mechanical constraint and the water availability at the optimal level or rather improve its mycorrhization level through the increases in the organic matter availability in the culture soil/medium which has been found here stimulating the short root production.

## 5. Conclusion

The success of the afforestation of Atlas cedar depends on the growth and performance of its seedlings, which are strongly linked to the adaptation rate of their root architecture opposite the hydromechanical properties of soil. In addition, the high medium strength limits the penetration ability of cedar root seedlings but stimulates its root branching at the optimal level. In contrast, high water availability inhibits the branching of cedar roots but stimulates its length. The combination of these two parameters at the optimal level accompanied with a high level of organic matter (sawdust) gives rise to long lateral roots and a high abundance of short roots. These characteristics may be very helpful in the mycorrhization process. On the contrary, the flexibility of the cedar root, at the anatomical scale, constitutes a strategy that cedar uses to escape the edaphic constraint as follows: the increase in tissue density in response to the high strength, the increment in the vascular bundle area to compensate the less root branching, and the adaptation of the phl/xyl ratio according to their growth strategies. In sum, all these hydromechanical proprieties must be taken into account depending on the soil of the afforestation region. However, our findings represent only a new contribution to the examination of these parameters' effects on a forest seedling species which consequently need more investigations at the level of the relationship between each parameter and the mycorrhizal response.

## Figures and Tables

**Table 1:** Mechanical and hydraulic characteristics of media.

Media	Acronym	*K* (cm s^−1^)	*n*	*ρ *_*d*_ (g cm^−3^)	*ρ *_sat_ (g cm^−3^)	WHC (%)
Hydroponic	Hyd	1.000 ± 0.0000^a^	1.000 ± 0.0000^a^	0.000 ± 0.000^a^	1.000 ± 0.0000^a^	100 ± 0.00^a^
Vermiculite	Ver	0.106 ± 0.0003^b^	0.951 ± 0.0139^b^	0.349 ± 0.013^b^	1.300 ± 0.002^b^	73.75 ± 1.87^b^
Sawdust	Saw	0.046 ± 0.0027^c^	0.839 ± 0.0406^c^	0.209 ± 0.016^b^	1.049 ± 0.043^a^	56.25 ± 1.87^c^
Vermiculite-gravel	VGr	0.022 ± 0.0007^d^	0.685 ± 0.0297^d^	1.089 ± 0.105^c^	1.773 ± 0.096^c^	57.50 ± 2.50^c^
Sand	San	0.016 ± 0.0003^e^	0.472 ± 0.0074^e^	1.480 ± 0.060^d^	1.952 ± 0.052^d^	27.50 ± 3.75^d^

*K*: permeability, *n*: porosity, *ρ*_*d*_: bulk density, *ρ*_sat_: density at saturation, and WHC: water-holding capacity. Mean ± SD followed by letters indicates significant differences (*P* < 0.05 LSD test).

**Table 2:** Water content of cedar seedlings and its various organs.

H_2_O %	Needles	Stem	Main root	Lateral roots
Hyd	263.96 ± 1.10^a^	2.18 ± 0.83^c^	579.85 ± 20.16^a^	13.64 ± 4.36^d^
Ver	198.29 ± 40.15^b^	176.58 ± 5.12^a^	264.28 ± 14.09^b^	436.58 ± 19.91^a^
Saw	134.07 ± 18.02^c^	125.49 ± 4.76^b^	213.29 ± 28.24^c^	457.03 ± 8.42^a^
VGr	299.23 ± 3.38^a^	195.25 ± 13.96^a^	209.38 ± 17.76^c^	346.98 ± 14.61^b^
San	263.64 ± 15.45^a^	190.32 ± 23.44^a^	247.61 ± 15.07^bc^	133.98 ± 25.12^c^

Mean ± SD followed by letters indicates significant differences (*P* < 0.05 LSD test). The acronyms of media are as designated as in Table 1.

**Table 3:** Dimensions of various root anatomical structures.

	Hyd	Ver	Saw	VGr	San
D. main root (*µ*m)	783.67 ± 2.56^d^	733.00 ± 14.77^e^	933.14 ± 17.85^b^	852.25 ± 6.43^c^	1614.73 ± 6.51^a^
W. epidermis (*µ*m)	25.18 ± 1.15^b^	14.00 ± 0.73^c^	27.17 ± 0.54^b^	25.74 ± 1.28^b^	37.55 ± 1.28^a^
W. cortex (*µ*m)	182.80 ± 1.13^d^	186.16 ± 4.37^d^	267.46 ± 5.52^b^	221.43 ± 6.27^c^	487.08 ± 7.84^a^
N. cortex cell	6.68 ± 0.15^cb^	7.18 ± 0.23^b^	6.56 ± 0.10^c^	6.50 ± 0.16^c^	7.98 ± 0.18^a^
W. vascular cylinder (*µ*m)	186.29 ± 3.92^b^	155.66 ± 4.00^c^	175.15 ± 3.85^b^	175.74 ± 1.55^b^	289.39 ± 3.07^a^
D. cortex cell (*µ*m)	27.74 ± 0.56^d^	26.38 ± 0.91^d^	41.12 ± 1.24^b^	34.74 ± 1.50^c^	62.07 ± 1.88^a^

*W*: width, *D*: diameter, and *N*: number. Mean ± SD followed by letters indicates significant differences (*P* < 0.05 LSD test). The acronyms of media are as designated as in Table 1.

**Table 4:** Root penetration rate of cedar seedlings on different media.

Medium	Penetration rate (mm/day)
Hyd	0.661 ± 0.036^a^
Ver	0.394 ± 0.051^b^
Saw	0.350 ± 0.077^bc^
VGr	0.271 ± 0.006^ce^
San	0.226 ± 0.010^e^

Mean ± SD followed by letters indicates significant differences (*P* < 0.05 LSD test). The medium acronyms are as designated as in Table 1.

**Table 5:** Pearson correlation coefficients between soil hydromechanical characteristics and root tissue width at 5% risk of the error.

		K (cm s^−1^)	*n*	*ρ *_*d*_ (g cm^−3^)	*ρ *_sat_ (g cm^−3^)	WHC (%)
Width (*µ*m)	Epidermis	−0.118	−0.705^*∗∗*^	0.537^*∗*^	0.431	−0.555^*∗*^
	Cortex	−0.425^*∗*^	−0.887^*∗∗*^	0.770^*∗∗*^	0.681^*∗∗*^	−0.860^*∗∗*^
	Vascular bundle	−0.157	−0.833^*∗∗*^	0.738^*∗∗*^	0.662^*∗∗*^	−0.704^*∗∗*^

K*:* permeability, *n*: porosity, *ρ*_*d*_: bulk density, *ρ*_sat_: density at saturation, and WHC: water-holding capacity. Bilateral correlation is significant at the *P* < 0.01 (^*∗∗*^) or *P* < 0.05 (^*∗*^) level.

**Table 6:** Pearson correlation coefficients between soil hydromechanical characteristics and seedling growth and root architecture at 5% risk of the error.

		K (cm s^−1^)	*n*	*ρ *_*d*_ (g cm^−3^)	*ρ *_sat_ (g cm^−3^)	WHC (%)
DW (mg)	Sd	0.795^*∗∗*^	0.509	−0.641^*∗∗*^	−0.682^*∗∗*^	0.569^*∗*^
	Nd	0.829^*∗∗*^	0.695^*∗∗*^	−0.748^*∗∗*^	−0.745^*∗∗*^	0.716^*∗∗*^
	St	0.996^*∗∗*^	0.590^*∗*^	−0.606^*∗*^	−0.590^*∗*^	0.784^*∗∗*^
	PR	−0.252	−0.593^*∗*^	0.534^*∗*^	0.484	−0.597^*∗*^
	LR	−0.536^*∗*^	−0.062	−0.151	−0.251	−0.338
N	SR	−0.691^*∗∗*^	−0.374	0.245	0.170	−0.569^*∗*^
	LR	−0.479	−0.460	0.289	0.191	−0.591^*∗*^
L (*µ*m)	PR	0.904^*∗∗*^	0.768^*∗∗*^	−0.768^*∗∗*^	−0.737^*∗∗*^	0.881^*∗∗*^
	LR	−0.471	0.153	−0.169	−0.170	−0.051
D (*µ*m)	PR	−0.357	−0.870^*∗∗*^	0.751^*∗∗*^	0.662^*∗∗*^	−0.822^*∗∗*^

DW: dry weight; N: number; *L* length; D: diameter; Sd: seedling; Nd needles; St stem; PR: main root; LR: lateral roots; SR: short roots; K: permeability; *n*: porosity; *ρ*_*d*_: bulk density; *ρ*_sat_: density at saturation; WHC: water-holding capacity. Bilateral correlation is significant at the *P* *<* *0.01* (^*∗∗*^) or *P* *<* *0.05* (^*∗*^) level.

## Data Availability

The data of this study are included within the article.
